# Supplementary material for: Structural Insights into the Distortion of the Ribosomal Small Subunit at Different Magnesium Concentrations
Source: Biomolecules. 2023 Mar 20;13(3):566. doi: 10.3390/biom13030566 (PMC10046523; doi:10.3390/biom13030566)
Supplement: Supplementary file 1 [file biomolecules-13-00566-s001.zip › Figure S1.pdf]

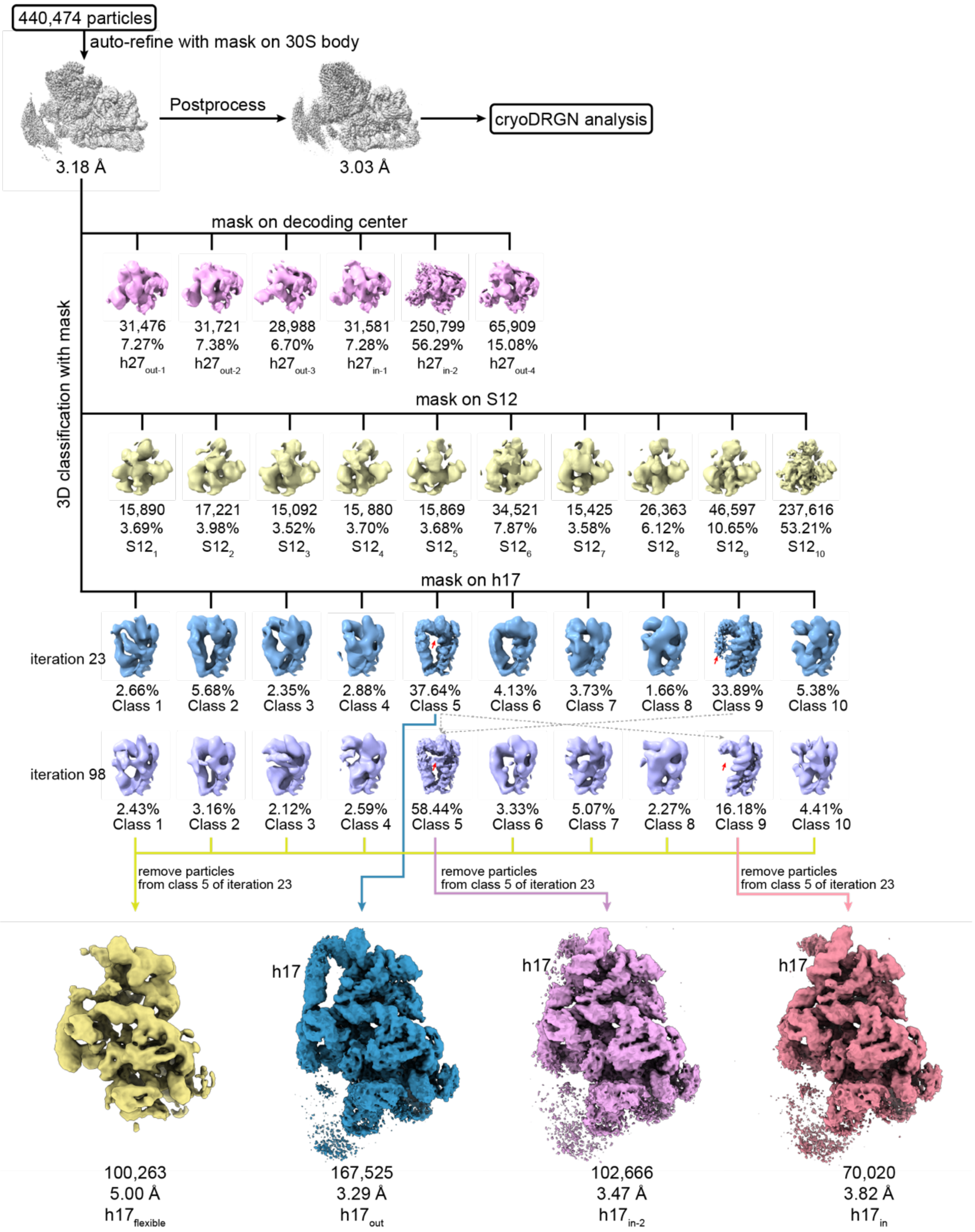

Figure S1. Structure analysis procedure of 30S from 1 mM Mg^{2+}. Particles filtered from 2D classification and initial 3D classification removing contaminations, bad ribosomal particles were subjected to refinement followed by 3D classification without angular alignment. Different masks on decoding center, S12 protein and h17 helix were used separately. CryoDRGN was used on all the particles based on refinement of the consensus reconstruction.
