# Supplementary material for: Structural Insights into the Distortion of the Ribosomal Small Subunit at Different Magnesium Concentrations
Source: Biomolecules. 2023 Mar 20;13(3):566. doi: 10.3390/biom13030566 (PMC10046523; doi:10.3390/biom13030566)
Supplement: Supplementary file 1 [file biomolecules-13-00566-s001.zip › Figure S2.pdf]

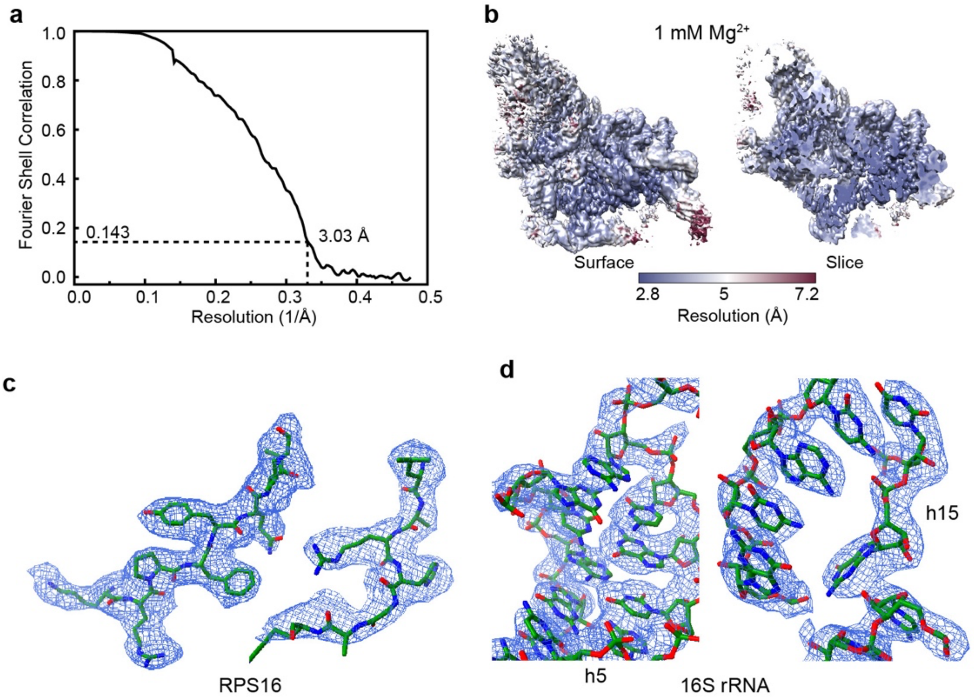

Figure S2. Quality of the map for 30S at 1 mM Mg^{2+}. a. The Fourier Shell Correlation (FSC) curve were calculated in Relion 3.1.0 and gold standard criteria with a value of 0.143 was used. **b.** The sharpened density map colored by local resolution in surface and slice views for the body of 30S under 1 mM Mg^{2+} concentration. **c, d.** Representative density for ribosomal protein (**c**) and 16S rRNA (**d**) showed the quality of the map.
