# Supplementary material for: Structural Insights into the Distortion of the Ribosomal Small Subunit at Different Magnesium Concentrations
Source: Biomolecules. 2023 Mar 20;13(3):566. doi: 10.3390/biom13030566 (PMC10046523; doi:10.3390/biom13030566)
Supplement: Supplementary file 1 [file biomolecules-13-00566-s001.zip › Figure S3.pdf]

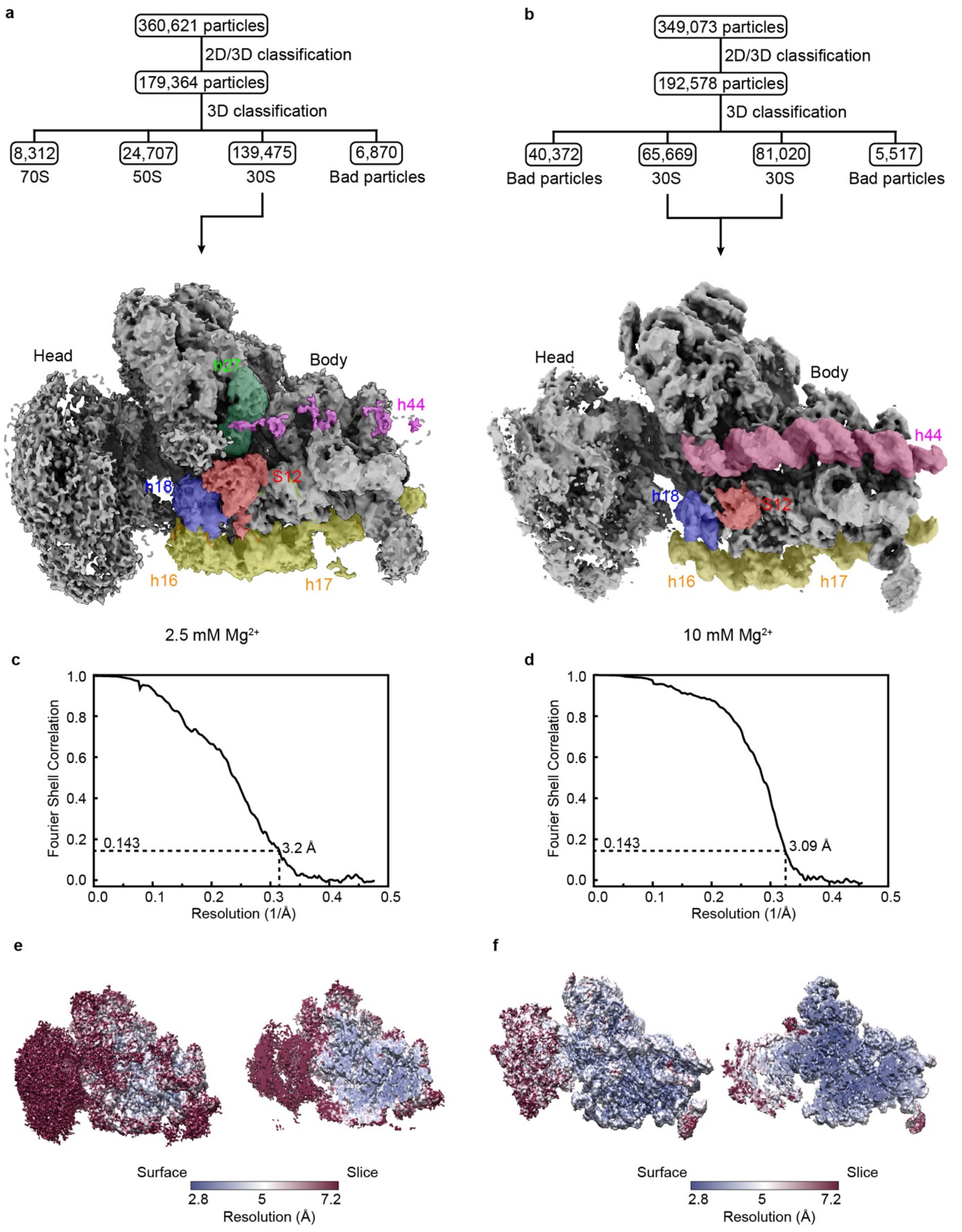

Figure S3. Overall maps of 30S under 2.5 and 10 mM Mg^{2+} condition. a, b. The 30S peaks of the sucrose gradients for BL21 DE3 at 2.5 mM Mg^{2+} (**a**) or 10 mM Mg^{2+} (**b**) were analyzed by cryoEM. Helix h16/h17, h18, h27, h44 and S12 were colored in yellow, blue, green, magenta and red, respectively. **c, d.** The Fourier Shell Correlation (FSC) curve for 2.5 mM (**c**)

and 10 mM (**d**) Mg^{2+} were calculated in Relion 3.1.0 and gold standard criteria with a value of 0.143 was used. **e, f.** The sharpened density map colored by local resolution in surface and slice views for the 30S under 2.5 (**e**) and 10 mM (**f**) Mg^{2+} concentration.
