# Supplementary material for: Structural Insights into the Distortion of the Ribosomal Small Subunit at Different Magnesium Concentrations
Source: Biomolecules. 2023 Mar 20;13(3):566. doi: 10.3390/biom13030566 (PMC10046523; doi:10.3390/biom13030566)
Supplement: Supplementary file 1 [file biomolecules-13-00566-s001.zip › Figure S4.pdf]

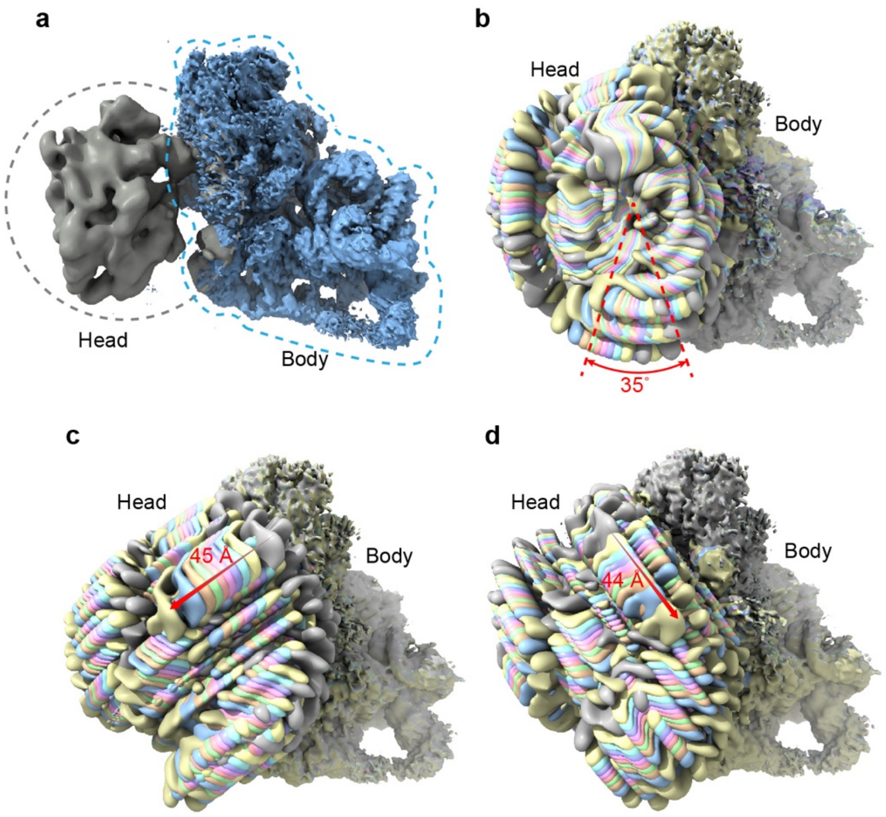

Figure S4. Low concentration of Mg^{2+} induce a large rotation of head in all directions. **a.** Multi-body refinement of particles for 30S at 1 mM Mg^{2+} with masks (dash line) on head (gray) or body (blue) part. **b.** Maps were aligned based on the body and rotation of head related to body was measured (red arrow). **c, d.** Same as a, but showing the rotation of head in other two directions (red arrows). The progressive colors of map represent different maps from volume 1 (grey) to 10 (yellow) in multi-body refinement.
