# Supplementary material for: Structural Insights into the Distortion of the Ribosomal Small Subunit at Different Magnesium Concentrations
Source: Biomolecules. 2023 Mar 20;13(3):566. doi: 10.3390/biom13030566 (PMC10046523; doi:10.3390/biom13030566)
Supplement: Supplementary file 1 [file biomolecules-13-00566-s001.zip › Figure S5.pdf]

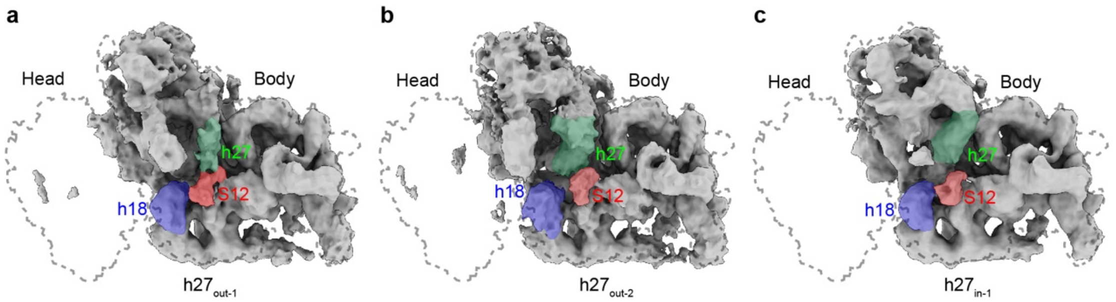

Figure S5. 30S under 1 mM Mg^{2+} showed different movements of h27. a-c. Classification of 30S with a mask on decoding center separate different conformations of h27. Helix h18, h27 and S12 were colored in blue, green and red, respectively. Dash line represent the mature 30S under 10 mM Mg^{2+} condition in this study.
