# Supplementary material for: Structural Insights into the Distortion of the Ribosomal Small Subunit at Different Magnesium Concentrations
Source: Biomolecules. 2023 Mar 20;13(3):566. doi: 10.3390/biom13030566 (PMC10046523; doi:10.3390/biom13030566)
Supplement: Supplementary file 1 [file biomolecules-13-00566-s001.zip › Figure S6.pdf]

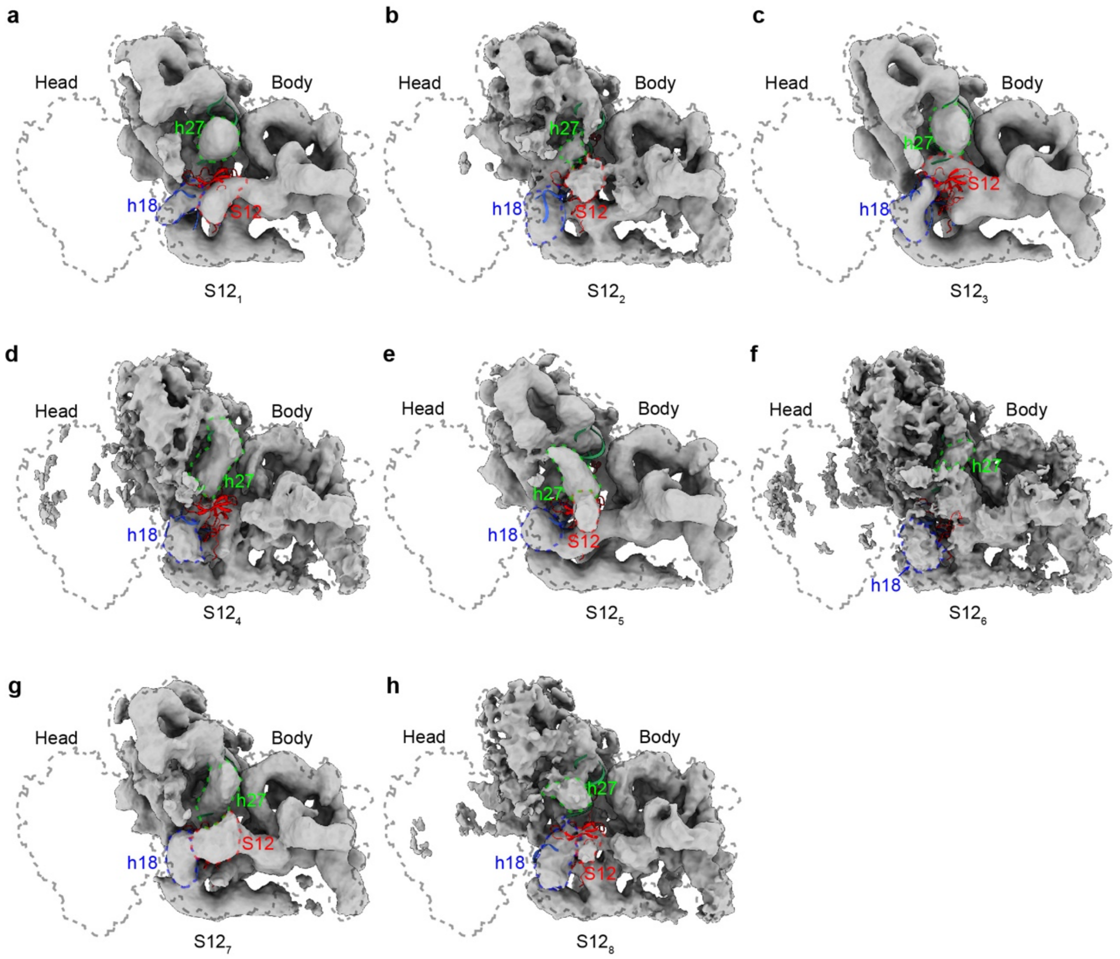

Figure S6. 30S under 1 mM Mg^{2+} showed a diverse S12 occupancy range. a-h. Classification of 30S with a mask on S12 separate different conformations of h27 and S12 in decoding center. The normal position of h18, h27 and S12 were shown as cartoon in blue, green and red, respectively. Gray dash line represents the 30S under 10 mM Mg^{2+} condition in this study. Colored dash line show the possible density for h18, h27 and S12 in each map.
