# Supplementary material for: Structural Insights into the Distortion of the Ribosomal Small Subunit at Different Magnesium Concentrations
Source: Biomolecules. 2023 Mar 20;13(3):566. doi: 10.3390/biom13030566 (PMC10046523; doi:10.3390/biom13030566)
Supplement: Supplementary file 1 [file biomolecules-13-00566-s001.zip › Figure S7.pdf]

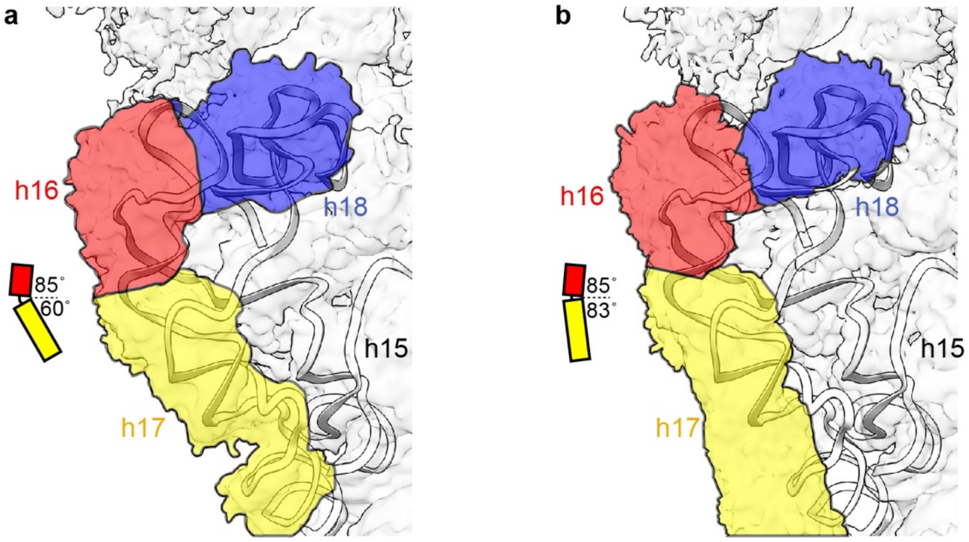

Figure S7. Movement of h17 does not affect the location of h16. a, b. Maps of $h17_{in}$ (**a**) and $h17_{out}$ (**b**) were represent as transparent and the region corresponding to h16, h17 and h18 were colored in red, yellow and blue, respectively. The location of these helices in a mature 30S (PDB: 7K00) were showed as cartoon in grey. Angles of h16 and h17 relative to the horizontal direction (dash line) were measured.
