# Supplementary material for: Structural Insights into the Distortion of the Ribosomal Small Subunit at Different Magnesium Concentrations
Source: Biomolecules. 2023 Mar 20;13(3):566. doi: 10.3390/biom13030566 (PMC10046523; doi:10.3390/biom13030566)
Supplement: Supplementary file 1 [file biomolecules-13-00566-s001.zip › Figure S8.pdf]

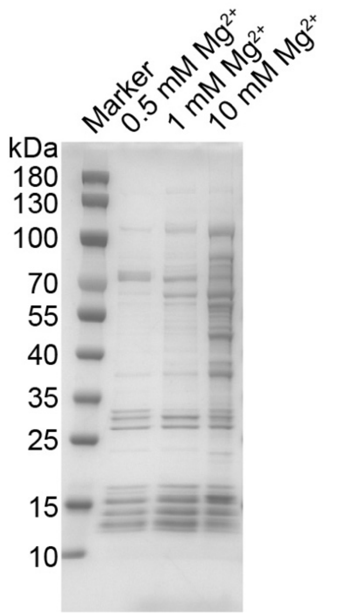

Figure S8. Low magnesium concentration disassociated the ribosomal proteins. Analysis of the ribosomal proteins for the 30S peak under 0.5, 1 and 10 mM Mg^{2+} by SDS-PAGE.
